# Assessing Determinants of Response to PARP Inhibition in Germline *ATM* Mutant Melanoma

**DOI:** 10.3390/ijms26157420

**Published:** 2025-08-01

**Authors:** Eleonora Allavena, Michela Croce, Bruna Dalmasso, Cecilia Profumo, Valentina Rigo, Virginia Andreotti, Irene Vanni, Benedetta Pellegrino, Antonino Musolino, Nicoletta Campanini, William Bruno, Luca Mastracci, Gabriele Zoppoli, Enrica Teresa Tanda, Francesco Spagnolo, Paola Ghiorzo, Lorenza Pastorino

**Affiliations:** 1 Department of Internal Medicine and Medical Specialties, University of Genoa, 16132 Genoa, Italy; eleonora.allavena@edu.unige.it (E.A.); william.bruno@unige.it (W.B.); gabriele.zoppoli@unige.it (G.Z.); lorenza.pastorino@unige.it (L.P.); 2 Cancer Genetics, IRCCS Ospedale Policlinico San Martino, 16132 Genoa, Italy; brunasamia.dalmasso@hsanmartino.it (B.D.); virginia.andreotti@hsanmartino.it (V.A.); irene.vanni@hsanmartino.it (I.V.); 3 Biotherapies, IRCCS Ospedale Policlinico San Martino, 16132 Genova, Italy; michela.croce@hsanmartino.it (M.C.); cecilia.profumo@edu.unige.it (C.P.); valentina.rigo@hsanmartino.it (V.R.); 4 Medical Oncology and Breast Unit, University Hospital of Parma, 43126 Parma, Italy; benedetta.pellegrino@unipr.it; 5 Medical Oncology, Breast & GYN Unit, IRCCS Istituto Romagnolo per lo Studio dei Tumori (IRST) “Dino Amadori”, 47014 Meldola, Italy; antonino.musolino@irst.emr.it; 6 Department of Medical and Surgical Sciences, University of Bologna, 40138 Bologna, Italy; 7 Unit of Pathological Anatomy, Department of Medicine and Surgery, University Hospital of Parma, 43126 Parma, Italy; nicoletta.campanini@unipr.it; 8 Anatomic Pathology, Department of Surgical Sciences and Integrated Diagnostics, University of Genova, 16132 Genoa, Italy; luca.mastracci@unige.it; 9 Anatomic Pathology, IRCCS Ospedale Policlinico San Martino, 16132 Genoa, Italy; 10 Medicina Interna a Indirizzo Oncologico, IRCCS Ospedale Policlinico San Martino, 16132 Genoa, Italy; 11 Skin Cancer Unit, IRCCS Ospedale Policlinico San Martino, 16132 Genova, Italy; enricateresa.tanda@hsanmartino.it (E.T.T.); francesco.spagnolo@unige.it (F.S.); 12 Department of Surgical Sciences and Integrated Diagnostics (DISC), University of Genoa, 16132 Genova, Italy

**Keywords:** melanoma, *ATM*, HRD, PARPi, germline variation

## Abstract

The ataxia–telangiectasia-mutated (ATM) protein plays a crucial role in the DNA damage response, particularly in the homologous recombination (HR) pathway. This study aimed to assess the impact of deleterious *ATM* variants on homologous recombination deficiency (HRD) and response to PARP inhibitors (PARPi) in melanoma patients, using a cell line established from melanoma tissue of a patient carrying the c.5979_5983del germline *ATM* variant. Despite proven loss of heterozygosity, lack of ATM activation, and HRD, our model did not show sensitivity to PARPi. We assessed the potential contribution of the Schlafen family member 11 (SLFN11) helicase, whose expression is inversely correlated with PARPi sensitivity in other cancers, to the observed resistance. The ATM mutant cell line lacked SLFN11 expression and featured hypermethylation-mediated silencing of the SLFN11 promoter. While sensitive to the ATR inhibitor (ATRi), the addition of ATRi to PARPi was unable to overcome the resistance. Our findings suggest that *ATM* mutational status and HRD alone do not adequately account for variations in sensitivity to PARPi in our model. A comprehensive approach is essential for optimizing the exploitation of DNA repair defects and ultimately improving clinical outcomes for melanoma patients.

## 1. Introduction

Remarkable advancements in melanoma treatment have been made in recent years with the introduction of immunotherapy and targeted therapies using BRAF and MEK inhibitors [1], significantly boosting patients’ overall survival. However, preclinical and clinical trials are needed and ongoing to explore new treatments (e.g., the use of Poly ADP-Ribose Polymerase inhibitors (PARPi)) and resistance mechanisms [2,3,4]. Homologous recombination (HR) deficient tumors are susceptible to platinum-based chemotherapy and to PARPi, which exploit DNA repair defects to induce synthetic lethality [5]. PARPi are currently used to treat patients with multiple types of HR-deficient tumors, specifically breast, ovarian, pancreatic, and prostate cancers [6,7,8,9]. Kim et al. showed that treatment of HR-DNA damage response (HR-DDR) mutated patient-derived xenograft models of melanoma with PARPi showed significant antitumor activity in vivo associated with increased apoptotic activity. Analyzing data from three major cohorts, they found that around 20% of melanomas harbor mutations in the HR-DDR pathway, paving the way to the application of PARPi to those melanomas with ataxia–telangiectasia-mutated (ATM) mutations, ranging from 2.4 to 7% of melanomas [10]. Fröhlich et al. showed that PARPi treatment could be a valuable therapy option for patients with melanoma resistant to MAPK inhibitors and exhibiting low basal ATM expression, supporting the concept that patients with BRCAness (not only BRCA mutated) and the homologous recombination deficiency (HRD) phenotype could benefit from PARPi therapy [11]. The ATM protein is a serine/threonine kinase involved in DDR. ATM is activated upon DNA double-strand breaks (DSBs) caused by ionizing radiation, oxidative stress, and indirectly, by DNA damage caused by UV radiation [12,13]. Biallelic loss-of-function (LOF) variants in the *ATM* gene result in ataxia–telangiectasia (AT) and predisposition to several hematologic and solid cancers [14]. Similarly, heterozygous carriers of *ATM* variants have an increased risk of several malignancies. For example, *ATM* is an established breast and pancreatic cancer predisposition gene, and pathogenic variants (PV) in *ATM* have also been implicated in susceptibility to gastric and prostate cancer, suggesting that the *ATM* tumor spectrum is likely broad [12]. Indeed, a multicentric international study on 2105 melanoma cases found an association between *ATM* germline heterozygous variants and melanoma risk [15]. Therefore, we propose *ATM*, which was previously identified as a melanoma genome-wide association study (GWAS) hit [16,17] and designated as a melanoma intermediate-risk gene [15]. Additionally, we provided experimental data supporting a double-hit inactivation by assessing ATM loss of expression and loss of heterozygosity (LOH) in melanoma tissue from germline carriers [18]. Among its pleiotropic functions, ATM activates cell cycle checkpoints by phosphorylating checkpoint kinase CHK2 and p53, ensuring that cells with damaged DNA do not progress through the cell cycle [19]. This checkpoint activation gives the cell time to repair DSB via processes such as HR. Therefore, loss of the ATM sensor may impair HR proficiency in these cells and serve as a biomarker of therapy response. In this study, we aimed to functionally assess the effects of a germline *ATM* pathogenic variant in melanoma by testing HRD and response to PARPi as a possible therapeutic approach. We also addressed molecular mechanisms determining PARPi sensitivity, analyzing the interplay between Schlafen family member 11 (SLFN11), a predictive biomarker of PARPi response, and ATM deficiency in melanoma, which is currently unknown.

## 2. Results

### 2.1. Melanoma ATM-Deficient Cell Lines Are HR Deficient

We selected the melanoma cell line (SPM63) established from melanoma tissue of a patient carrying the c.5979_5983del germline *ATM* PV (in which we previously characterized LOH and absence of protein expression) [18] and confirmed its lack of ATM activation by testing p-ATM expression before and after irradiation. As a control we used the SPM59 melanoma cell line, with no variants in *ATM* (*ATM*-wild-type (WT) cell line), in which we observed very high levels of p-ATM (Supplementary Figure S1) and the SK-CO-1 colorectal cancer cell line, which carries a homozygous variant in *ATM* (c.2251-10T>G) and lacks functional ATM, as previously described [20]. We then analyzed and compared RAD51 foci formation in the selected cell lines as a marker of HRD [8]. The *ATM*-mutated SPM63 and SK-CO-1 cell lines exhibited impaired RAD51 foci formation, with only 5% of geminin-positive cells. Since geminin acts as a checkpoint in the cell cycle, preventing premature or excessive DNA replication and ensuring the orderly progression of cells through the S and G2 phases, this indicates impairment of the homologous recombination repair (HRR) pathway, resulting in HRD (Supplementary Figure S2). In contrast, the SPM59 cell line demonstrated a high percentage of RAD51 foci formation, with 30% of geminin-positive cells, indicating that this cell line is HR proficient.

### 2.2. ATM-Deficient Melanoma Cells Do Not Respond to PARPi Despite HRD

**Figure 1:**
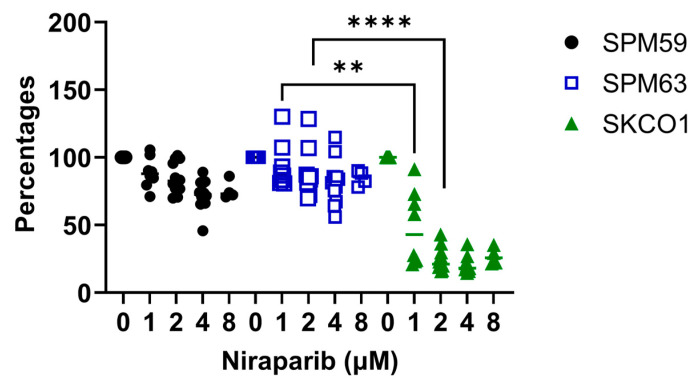
Cell viability expressed as percentages of OD values normalized to the control of SPM59, SPM63, and SK-CO-1 cells treated with increasing doses of niraparib for 72 h.** *p* < 0.02, **** *p* < 0.001).

We evaluated the effect of PARPi on cell proliferation by treating cells with increasing doses of niraparib (up to 8 µM). The SPM63 cell line, as well as SPM59, showed no sensitivity to niraparib, in contrast to SK-CO-1, which exhibited high sensitivity even at low concentrations (Figure 1).

**Figure 2:**
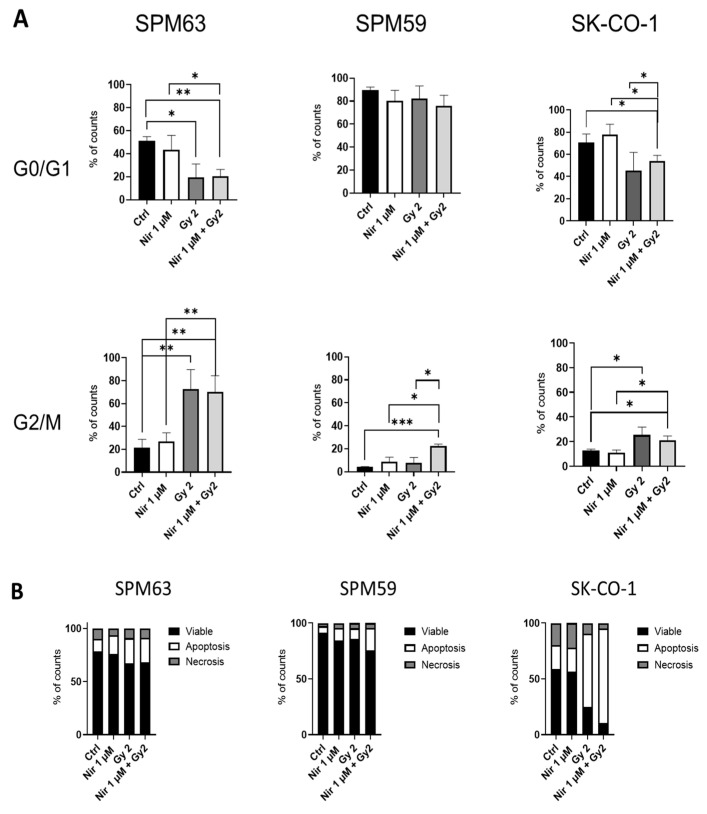
SPM63, SPM59, and SK-CO-1 treated for 24 h with niraparib (Nir) and/or irradiation (2 Gy) and analyzed for (**A**) cell cycle and (**B**) apoptosis after 7 days. Apoptotic cells are defined as Annexin Vpos Propidium Iodide (PI)pos/neg; necrotic cells are Annexin Vneg PIpos; viable cells are Annexin Vneg PIneg. Percentages of counts are shown. * *p* < 0.05, ** *p* < 0.02, *** *p* < 0.01.

Irradiation, rather than treatment with PARPi, resulted in a more pronounced impact on the cell cycle in SPM63 compared to SK-CO-1, blocking a substantial fraction of SPM63 cells in the G2/M phase while decreasing the proportion of those in the G0/G1 phase (Figure 2A). In the SPM59 cell line, only the combination of irradiation and niraparib increased the proportion of cells in the G2/M phase, without a significant effect on the G0/G1 phase. Regarding apoptosis, we observed an increased percentage of apoptotic SK-CO-1 cells in response to irradiation, which was slightly enhanced by adding niraparib. The ATM-deficient SPM63 cell line exhibited a slightly higher percentage of apoptotic cells after irradiation compared to WT- SPM59, with no modification from the addition of the drug (Figure 2B).

### 2.3. SLFN11 Inactivation Correlates with Resistance to PARPi

**Figure 3:**
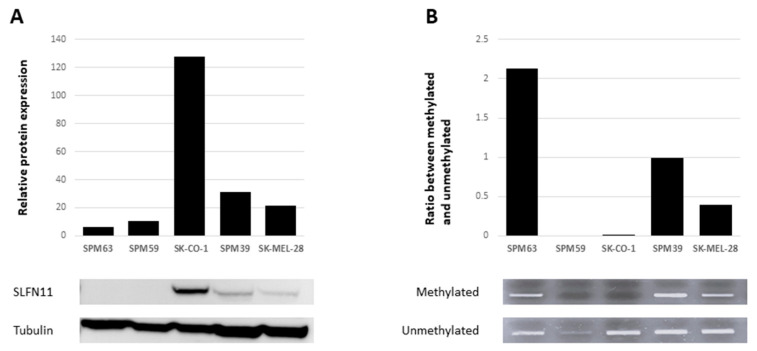
Correlation between SLFN11 expression and promoter methylation. (**A**) Baseline expression of SLFN11 by Western blotting on multiple cell lines. Densitometric analysis of a representative assay (below). (**B**) Methylation of the *SLFN11* promoter analyzed by methylation-specific PCR (MSP) in multiple cell lines. Tapestation densitometric analysis, expressed as the ratio of methylated DNA to unmethylated DNA. Below agarose gel image for the corresponding cell lines.

Since Schlafen family member 11 (SLFN11) has been proposed as a predictive biomarker of PARPi response, we assessed its potential role in ATM-deficient cell lines SPM63 and SK-CO-1, as well as in two additional WT melanoma cell lines, SPM39 and SK-MEL-28. SLFN11 was expressed in SK-CO-1, SPM39, and SK-MEL-28, while it was absent in SPM63 and SPM59 (Figure 3A), correlating with the observed PARPi response. Assuming that loss of SLFN11 expression may be associated with promoter methylation, we performed a methylation analysis of its promoter using methylation-specific PCR (MSP). Promoter methylation often leads to gene silencing, a phenomenon observed in various types of cancer. By using MSP, we can specifically detect the methylation status of the *SLFN11* promoter, which helps us understand whether methylation is responsible for the reduced expression of SLFN11 in our samples [21,22]. *SLFN11* promoter was found to be methylated in the ATM-deficient SPM63 cell line, unmethylated in SPM59 and SK-CO-1, and partially methylated in SPM39 and SK-MEL-28 cell lines (Figure 3B). These findings suggest an association between reduced/loss of SLFN11 expression and promoter region methylation in melanoma cell lines, apart from the SPM59 cell line, where other epigenetic mechanisms involved in suppressing SLFN11 expression may be hypothesized.

### 2.4. ATM-Deficient Melanoma Cells Show Sensitivity to ATRi

**Figure 4:**
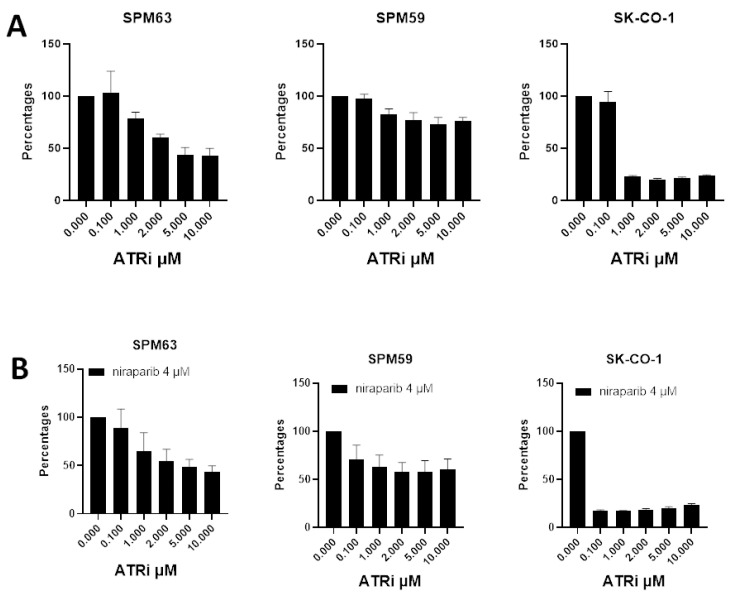
Cell viability expressed as percentages of the OD values normalized to the control of SPM63, SPM59, and SK-CO-1 treated with (**A**) different concentrations of ATRi alone and together with (**B**) a representative single dose of niraparib (4 μM).

We evaluated the effects of ATRi, either alone or in combination with niraparib, to explore potential mechanisms by which the SPM63 ATM- and SLFN11-deficient primary melanoma cell line could evade PARPi-induced cell death. Our experiments showed that the SPM63 ATM-deficient cell line exhibited greater sensitivity to ATRi compared to SPM59, particularly at higher concentrations (5–10 µM); however, this effect was less pronounced than that observed in the SK-CO-1 cell line (Figure 4A) [20]. The addition of the PARPi did not enhance the effect of ATRi at any of the tested concentration combinations; a representative experiment with the 4 µM niraparib is shown (Figure 4B).

## 3. Discussion

PARP inhibitors (PARPi) are a class of targeted cancer therapies designed to exploit weaknesses in the DNA repair pathways of cancer cells. Although they are primarily recognized for their efficacy in treating BRCA-mutated breast and ovarian cancers, recent studies are increasingly uncovering their potential applications in melanoma treatment [2,10]. Ongoing clinical trials are examining the therapeutic potential of PARP inhibitors for melanoma patients, especially those with particular genetic alterations (HR Mutation—sourced from ClinicalTrials.gov, https://clinicaltrials.gov/ (accessed on 17 January 2025)) [23]. These trials seek to determine which melanoma patient subgroups are most likely to benefit from this treatment approach. Incorporating functional assays to evaluate PARP inhibitor efficacy in vitro may provide valuable insights and enable personalized therapy based on the distinct molecular features of each tumor. To this end, we investigated the responsiveness of our ATM-mutant melanoma model to PARP inhibition, beginning with an assessment of HRD. Our results showed that the SPM63 and SK-CO-1 cell lines, both carrying *ATM* mutations, exhibit markedly reduced RAD51 foci formation, indicating a compromised HRR pathway. In contrast, the SPM59 ATM WT cell line exhibited diffuse RAD51 foci formation, confirming its HRR proficiency. These HRR differences between ATM-deficient and ATM WT cells validate the use of these melanoma cell lines as appropriate models for investigating the effects of PARP inhibition.

After assessing the proliferation of these cell lines following treatment with niraparib, we observed that the sensitivity to PARPi did not differ between the ATM-deficient SPM63 cell line and the WT SPM59. Conversely, SK-CO-1 showed sensitivity to PARPi even at low concentrations, consistent with the findings previously reported by Wang et al. [20]. Given that tumors with *ATM* mutations or other DNA repair deficiencies are especially sensitive to the combined effects of PARP inhibitors and irradiation due to their impaired ability to repair damage induced by both treatments [24], we examined cell cycle progression and apoptosis following treatment with PARP inhibitors alone and in combination with irradiation. Our results showed that irradiation, rather than niraparib, influenced the cell cycle in the SPM63 and SK-CO-1 cell lines. Specifically, SPM63 cells displayed a greater accumulation in the G2/M phase following irradiation, accompanied by a reduction in the G0/G1 phase compared to SK-CO-1 cells. This indicates irradiation-induced G2/M phase arrest, a typical cellular response to DNA damage as cells prepare for mitosis. In contrast, the SPM59 cell line exhibited an increased G2/M phase only when treated with the combination of irradiation and niraparib, with no significant changes observed in the G0/G1 phase.

This variable response likely reflects differences in the inherent DNA repair capabilities of the cell lines studied, possibly due to the presence of functional ATM in SPM59. Apoptosis analysis showed an increased proportion of apoptotic cells in SK-CO-1 following irradiation, an effect that was slightly amplified by the addition of niraparib. The slightly increased apoptosis observed in the ATM-deficient SPM63 cell line indicates a possible heightened sensitivity to stress in the absence of functional ATM. Importantly, this response was not further amplified by drug treatment, unlike in the ATM-functional SPM59 cell line, which showed elevated p-ATM phosphorylation, supporting the observed effect (Supplementary Figure S1). Despite exhibiting HRD, the SPM63 cell line remains resistant to PARP inhibitors (PARPi), as demonstrated by proliferation, cell cycle, and apoptosis assays. Instead, irradiation—not PARPi—induces changes in cell cycle progression and apoptosis in SPM63. In order to explore a potential resistance mechanism, we examined the role of SLFN11, a DNA repair mediator that sensitizes cells to DNA-damaging agents by blocking DNA replication in repair-deficient cells. In ATM-deficient cancer cells, SLFN11 can enhance sensitivity to such agents, underscoring its potential as both a therapeutic target and a biomarker in cancer treatment [21,25]. Synergism between PARPi and ATR inhibitors has been described in ATM-deficient cancer cells [26]. Moreover, adding ATRi can overcome the resistance to PARPi in SLFN11-negative cells. However, to the best of our knowledge, data on PARPi resistance and SLFN11 expression in melanoma are unavailable.

In our cell lines, SLFN11 expression showed a good correlation with resistance to PARPi. Promoter methylation, already described in other cell line models [27], was hypothesized to be a mechanism of SLFN11 inactivation in our SPM63 cells as well. Further studies are needed to confirm the role of SLFN11 in this setting, especially its influence on treatment outcomes and its potential as a biomarker for guiding therapy in melanomas with impaired DNA repair mechanisms.

Studies have shown that ATRi can potentially overcome PARPi resistance caused by SLFN11 loss, as SLFN11-deficient cells rely on ATR signaling to survive under PARPi treatment [28]. The SPM63 cell line exhibited greater sensitivity to ATR inhibition than SPM59, suggesting that ATM deficiency increases reliance on ATR-mediated DNA damage response pathways. While we observed a strong anti-proliferative effect when PARPi were added to ATRi at low concentrations in the SK-CO-1 cell line, in our melanoma model, the addition of ATRi to PARPi was unable to overcome resistance in our model, indicating that combinatorial approaches may not be effective in the context of ATM deficiency in melanoma cells. Our study has several limitations. First, our experiments were conducted on a limited number of primary melanoma cell lines, and response to PARPi was described in only one ATM-deficient melanoma cell line established from a patient carrying a germline PV. Additionally, we relied solely on baseline DNA and RNA sequencing data, as the previously used cell lines had undergone whole-exome sequencing and RNA sequencing [29]. However, no data are currently available on the activation of the downstream DDR pathway resulting from ATM loss, either before or after treatments.

These results highlight the complexity of DDR mechanisms and underscore the need for further exploration of tailored treatment strategies for different genetic backgrounds in cancer.

## 4. Materials and Methods

### 4.1. Cell Culture

Human melanoma primary cell lines (SPM63, SPM59, and SPM39) were obtained from patients enrolled at the IRCCS Ospedale Policlinico San Martino under local Institutional Review Board (IRB) approved protocols (CER Liguria: 046REG2017), as previously described [18]. SPM63 was established in our laboratory from fresh melanoma tissue of a patient carrying the c.5979_5983del germline *ATM* variant, while SPM59 and SPM39 were established, under the same condition, from two sporadic melanoma patients with no germline or somatic pathogenic variants in *ATM*, as determined by whole-exome sequencing, as previously reported [29]. Therefore, they were included as *ATM*-WT controls. Cell lines were cultured in RPMI medium supplemented with 10% fetal bovine serum (FBS), 2% Glutamine, 1% HEPES, and 1% sodium pyruvate.

Additionally, we used two commercial cell lines: SK-CO-1 (RRID:CVCL_0626), a colorectal cancer cell line that carries a homozygous variant in the *ATM* gene (c.2251-10T>G) and lacks protein expression, and SK-MEL-28 (RRID:CVCL_0526), a melanoma cell line ATM-WT. The SK-CO-1 was selected because of its sensitivity to PARPi [20]. Both cell lines were cultured in DMEM supplemented with 10% (FBS) and 2% Glutamine.

### 4.2. Immunostaining of RAD51 Foci

In response to double-strand DNA damage, activation of the homologous recombination repair (HRR) pathway leads to the formation of RAD51 foci. The RAD51 assay is a functional and dynamic immunofluorescence-based method used to evaluate HRD, performed on formalin-fixed, paraffin-embedded (FFPE) tissue samples [30,31], allowing for the assessment of HR proficiency.

For this assay, 10 × 10^6^ cells were pre-embedded in low-melting-point agarose gel, followed by fixation and paraffin embedding using the same protocol applied to tissue samples [32].

The following primary antibodies were used for immunofluorescence staining: rabbit anti-RAD51 (Abcam, Cambridge, UK, Cat# ab133534, RRID: AB_2722613; 1:1000), mouse anti-geminin (Novocastra, Newcastle upon Tyne, UK, Cat# NCL-L; 1:60), rabbit anti-geminin (Proteintech, Rosemont, IL, USA, Cat# 10802-1-AP, RRID: AB_2110945; 1:400), and mouse anti-phospho-γH2AX (Millipore, Billerica, MA, USA, Cat# 05-636; 1:200). Secondary antibodies included goat anti-rabbit Alexa Fluor 568 (Invitrogen, Waltham, MA, USA; 1:500), goat anti-mouse Alexa Fluor 488 (Invitrogen; 1:500), donkey anti-mouse Alexa Fluor 568 (Invitrogen; 1:500), and goat anti-rabbit Alexa Fluor 488 (Invitrogen; 1:500). Nuclei were counterstained with DAPI using ProLong™ Gold Antifade Mountant (Invitrogen) prior to microscopy. Immunofluorescence staining was performed at the University Hospital of Parma, following the procedures described in [30].

Biomarker levels were quantified in FFPE patient tumor samples by calculating the percentage of geminin-positive cells exhibiting five or more nuclear foci. Geminin, a key regulator of cell cycle progression, serves as a marker for cells in the S/G2 phase [33]. Scoring was conducted on live-cell images using a 100× oil-immersion objective lens. For each sample, 100 geminin-positive cells were analyzed from at least three representative regions. Samples were excluded from analysis if they exhibited low endogenous DNA damage—defined as fewer than 25% of geminin-positive cells with gH2AX foci—or contained fewer than 40 geminin-positive cells, indicating an insufficient number of tumor cells in the S/G2 phase. Each sample was scored twice by the same investigator on two technical replicates, using the Nikon TiE microscope at the University Hospital of Parma. In cases of inconsistent results, samples were re-stained and re-scored by the same investigator at the same facility. Tumors were classified as homologous recombination (HR) deficient (≤10%) or HR proficient (>10%) based on a predefined RAD51 score threshold of 10% [34].

### 4.3. Proliferation Assay

To assess sensitivity to the PARP inhibitor, we plated 7000 SPMs or SK-CO-1 cells per well in 96-well plates and treated them the following day with increasing concentrations of niraparib (MK-4827; 1–8 μM) for 72  h. To evaluate the effects of niraparib in combination with ATRi, we plated the above-mentioned cells and treated them with increasing concentrations of niraparib (1–4 μM) and ATRi (Ceralasertib AZD6738; 0.1–10 μM), either alone or in combinations, starting the day after seeding the cells for 72 h. We examined cell viability in the presence of fixed niraparib concentrations with different ATRi doses. Cell viability was assessed using the CellTiter 96 AQueous One Solution Cell Proliferation Assay (MTS Assay; Promega Corp., Madison, WI, USA) according to the manufacturer’s instructions. OD values at 490 nm were normalized to the control cells’ OD and represented as percentages.

### 4.4. Apoptosis and Cell Cycle Analysis

Briefly, SPM59 and SPM63 cells were seeded at a density of 600,000 cells per Petri dish, while SKCO-1 cells were seeded at 300,000 cells per dish. Cells in the logarithmic growth phase were treated with 1 μM niraparib one hour before irradiation. Twenty-four hours after exposure to 2 Gy radiation, cells were trypsinized, counted, and replated at a density of 200,000 cells per well in 24-well plates. Treatment regimens were selected based on published literature [35,36]. Six days folloing the initial niraparib treatment, cells were harvested for apoptosis and cell cycle analysis. Apoptosis was detected using Annexin V-fluorescein isothiocyanate (FITC) and Propidium Iodide, according to the manufacturer’s instructions (eBioscience, Thermo Fisher Scientific, Waltham, MA, USA). The expression of Annexin V with or without Propidium Iodide (PI) positivity (PI is a DNA-binding dye that only enters cells with a compromised membrane) allowed us to define apoptotic cells, while PI positivity in the absence of Annexin V defined necrotic cells. Double-negative cells were considered viable [37]. The samples were analyzed using a FACScan Flow cytometer and CellQuest software (version 7.5.3) (BD Biosciences, San Jose, CA, USA). For the cell cycle analysis, cells were harvested, washed in PBS, permeabilized in cold 70% ethanol, and incubated overnight at 4 °C in the dark. After washing with PBS, the cells were incubated in 1 mL of PI staining solution for 30 min at room temperature in the dark. The cell cycle was determined using a flow cytometer (FACScan; Becton Dickinson, Milan, Italy) and analyzed with ModFit LT v3.0 software (Beckman Coulter, Indianapolis, IN, USA).

### 4.5. Western Blot

Total proteins were extracted from both untreated and irradiated cell lines using cOmplete Lysis-M buffer (Roche, Mannheim, Germany) supplemented with protease inhibitors (Roche, Cat# 04719956001) and phosphatase inhibitors (Roche, Cat# 04906845001). Protein lysates were separated under reducing conditions on NuPAGE 4–12% Bis-Tris gels (Invitrogen, Cat# NP0335BOX). Western blotting was performed following standard protocols using the following primary antibodies: rabbit anti-pATM (S1981) (Abcam, Cat# ab81292, RRID:AB_1640207), rabbit anti-SLFN11 (Abcam, Cat# ab121731, RRID:AB_11128477), and rabbit anti-β-tubulin (Abcam, Cat# ab21058, RRID:AB_727045). An anti-rabbit HRP-conjugated secondary antibody (Agilent, Santa Clara, CA, USA, Cat# P0448, RRID:AB_2617138) was used for detection.

Immunoreactive bands were visualized using ECL Prime (GE Healthcare, Chicago, IL, USA, Cat# RPN2232) and a chemiluminescence gel documentation system (MINI HD, UVITEC, Cambridge, UK). Densitometric analysis was performed by calculating the ratio of SLFN11 to β-tubulin signal intensities.

### 4.6. DNA Extraction and Methylation

Genomic DNA from cell culture was extracted with the DNeasy Blood & Tissue Kit (Qiagen, Hilden, Germany) according to the manufacturer’s instructions. Bisulfite conversion was performed using the EZ DNA methylation kit (Zymo Research Corp, Irvine, CA, USA). PCR was performed using the following primers: 5′-TTTGGAAGGTGGGATCGTAGGTATC-3′ (forward methylated primer) and 5′-ACCCAAACAACTATCGACTCCTACG-3′ (reverse methylated primer); 5′-TATTTGGAAGGTGGGATTGTAGGTATT-3′ (forward unmethylated primer) and 5′ AAACCCAAACAACTATCAACTCCTACA-3′ (reverse unmethylated primer) [38], 1 µL of converted DNA and FastStart™ Taq DNA Polymerase (Roche Diagnostics GmbH, Mannheim, Germany). The PCR product was run on a 2% agarose gel containing Ethidium Bromide. The purity and quantity of DNA size fragments were assessed using the Agilent High Sensitivity DNA Analysis Kit (Agilent Technologies, Santa Clara, CA, USA) on the Tapestation 2200 instrument (Agilent Technologies). DNA concentrations determined by TapeStation analysis were used to quantify methylation by calculating the ratio of methylated to unmethylated DNA, providing a relative measure of methylation levels in each sample.

### 4.7. Statistical Analysis

All the comparisons between control and treatment were analyzed by a two-sided *T*-test for independent samples. *p* values lower than 0.05 were considered significant: * *p* < 0.05, ** *p* < 0.02, *** *p* < 0.01, **** *p* < 0.001. Analyses were performed using PRISM v 9.4 (GraphPad, La Jolla, CA, USA).

## 5. Conclusions

This study underscores the complexity of targeting DNA damage repair (DDR) pathways in melanoma, particularly in the context of ATM deficiency. While ATM-mutant melanoma cells demonstrate features of homologous recombination deficiency (HRD), they do not consistently respond to PARP inhibition, as shown by the resistance of the SPM63 cell line. The role of SLFN11 emerges as a potential factor influencing this resistance, with its inactivation possibly contributing to poor PARPi sensitivity. Moreover, the combination of PARPi and ATR inhibitors was ineffective in overcoming resistance in ATM-deficient cells, highlighting the limitations of current combinatorial strategies. These findings emphasize that *ATM* mutational status alone is insufficient as a predictive biomarker for PARPi response in melanoma. A more comprehensive approach, integrating multiple molecular indicators such as SLFN11 expression, will be critical for personalizing DDR-targeted therapies and improving clinical outcomes in melanoma patients.

## Data Availability

The data are available upon reasonable request to the authors.

## Supplementary Materials

The following supporting information can be downloaded at https://www.mdpi.com/article/10.3390/ijms26157420/s1.

## Abbreviations

The following abbreviations are used in this manuscript:

AT	Ataxia–telangiectasia
ATM	Ataxia–telangiectasia-mutated
ATRi	ATR inhibitor
DDR	DNA damage response
DSB	DNA double-strand breaks
FFPE	Formalin-fixed paraffin-embedded
GWAS	Genome-wide association study
HR	Homologous recombination
HRD	Homologous recombination deficiency
HRR	Homologous recombination repair
LOF	Loss-of-function
LOH	Loss of heterozygosity
MSP	Methylation-specific PCR
PV	Pathogenic variants
PARPi	Poly ADP-Ribose Polymerase inhibitors
SLFN11	Schlafen family member 11
