# Supplementary material for: Assessing Determinants of Response to PARP Inhibition in Germline ATM Mutant Melanoma
Source: Int J Mol Sci. 2025 Aug 1;26(15):7420. doi: 10.3390/ijms26157420 (PMC12346925; doi:10.3390/ijms26157420)
Supplement: Supplementary file 1 [file ijms-26-07420-s001.zip › ijms-3764870-supplementary.pdf]

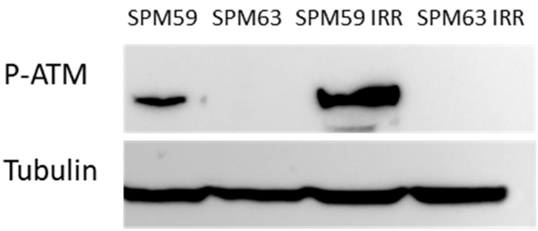

Figure S1: ATM inactivation in the SPM63 melanoma model. p-ATM protein expression by Western Blot (WB) in SPM59 and SPM63 before and after irradiation.

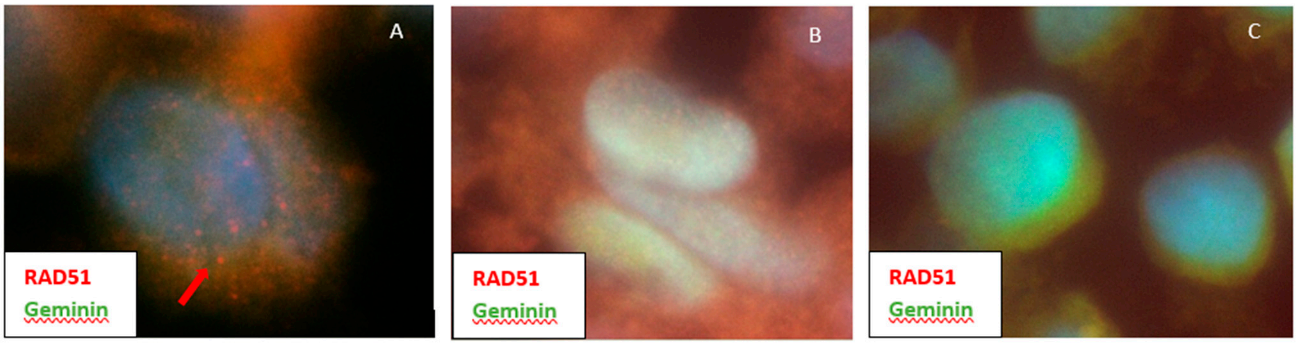

Figure S2: RAD51 nuclear foci detected by immunofluorescence (IF) in geminin-positive cells. A) IF of RAD51 in SPM59 cell line; B) IF of RAD51 in SPM63 cell line; C) IF of RAD51 in SK-CO-1 cell line (The red arrow points at RAD51 foci in geminin-positive cells).
